# Triple-Negative Breast Cancer circRNAome Reveals Hsa_circ_0072309 as a Potential Risk Biomarker

**DOI:** 10.3390/cancers14133280

**Published:** 2022-07-05

**Authors:** Leandro Magalhães, André M. Ribeiro-dos-Santos, Rebecca L. Cruz, Kivvi Duarte de Mello Nakamura, Rafael Brianese, Rommel Burbano, Sâmio Pimentel Ferreira, Ewaldo Lúcio Foro de Oliveira, Ana Karyssa Mendes Anaissi, Márcia Cristina de Sousa Nahúm, Samia Demachki, Amanda F. Vidal, Dirce Maria Carraro, Ândrea Ribeiro-dos-Santos

**Affiliations:** 1 Laboratory of Human and Medical Genetics, Postgraduate Program of Genetics and Molecular Biology, Institute of Biological Sciences, Federal University of Pará, Belém 66075-110, Brazil; leandromag@ufpa.br (L.M.); ribeia01@nyu.edu (A.M.R.-d.-S.); rebecca.cruz@icb.ufpa.br (R.L.C.); amanda.vidal@pq.itv.org (A.F.V.); 2 Genomic and Molecular Biology Group, International Research Center/CIPE, A.C. Camargo Center, São Paulo 01508-010, Brazil; kdmnakamura@accamargo.org.br (K.D.d.M.N.); rcbrianese@accamargo.org.br (R.B.); dirce.carraro@accamargo.org.br (D.M.C.); 3 Molecular Biology Laboratory, Ophir Loyola Hospital, Belém 66063-240, Brazil; rommel@ufpa.br; 4 Department of Clinical Oncology, Ser Clínica Oncológica, Belém 66035-265, Brazil; samiopimentel@ig.com.br (S.P.F.); ewaldooliveira@uol.com.br (E.L.F.d.O.); 5 Postgraduate Program of Oncology and Medical Sciences, Center of Oncology Research, Federal University of Pará, Belém 66073-000, Brazil; ana.anaissi@ebserh.gov.br (A.K.M.A.); gsnahum@gmail.com (M.C.d.S.N.); demachki@ufpa.br (S.D.); 6 Environmental Genomics Laboratory, Vale Institute of Technology, Belém 66055-090, Brazil; 7 National Institute of Science and Technology in Oncogenomics and Therapeutic Innovation (INCITO), A.C. Camargo Center, São Paulo 01508-010, Brazil

**Keywords:** circular RNAs, triple-negative breast cancer, gene regulation, biomarkers

## Abstract

**Simple Summary:**

Triple Negative Breast Cancer (TNBC) is a highly aggressive type of cancer that lacks biomarkers for its early discovery, leading to overall poor prognosis after its diagnosis. Circular RNAs (circRNAs) are a new class of regulatory RNAs and are promising biomarkers for several human diseases, including TNBC. In this study, we profiled the expression of all circRNAs present in TNBC in order to identify new biomarkers for this disease and it was possible to observe that 16 were deregulated, among them hsa_circ_0072309. In two distinct sets of samples, hsa_circ_0072309 was able to distinguish TNBC from healthy controls, making it a promising risk biomarker for this disease. Additionaly, since circRNAs are known to interact with RNA-Binding Proteins (RBPs), we investigated its probable function in this cancer and found that by interacting with such RBPs, this circRNA is acting in several cancer-related biological pathways. Recognizing these differentially expressed circRNAs and identifying their role can lead to a better understanding of dysregulated pathways in TNBC and ultimately allow the development of personalized therapies in this molecular subtype of breast cancer.

**Abstract:**

Circular RNAs (circRNAs) are a class of long non-coding RNAs that have the ability to sponge RNA-Binding Proteins (RBPs). Triple-negative breast cancer (TNBC) has very aggressive behavior and poor prognosis for the patient. Here, we aimed to characterize the global expression profile of circRNAs in TNBC, in order to identify potential risk biomarkers. For that, we obtained RNA-Seq data from TNBC and control samples and performed validation experiments using FFPE and frozen tissues of TNBC patients and controls, followed by in silico analyses to explore circRNA-RBP interactions. We found 16 differentially expressed circRNAs between TNBC patients and controls. Next, we mapped the RBPs that interact with the top five downregulated circRNAs (hsa_circ_0072309, circ_0004365, circ_0006677, circ_0008599, and circ_0009043) and hsa_circ_0000479, resulting in a total of 16 RBPs, most of them being enriched to pathways related to cancer and gene regulation (e.g., AGO1/2, EIF4A3, ELAVL1, and PTBP1). Among the six circRNAs, hsa_circ_0072309 was the one that presented the most confidence results, being able to distinguish TNBC patients from controls with an AUC of 0.78 and 0.81, respectively. This circRNA may be interacting with some RBPs involved in important cancer-related pathways and is a novel potential risk biomarker of TNBC.

## 1. Introduction

Circular RNAs (circRNAs) are a class of non-coding RNAs characterized by their 5′ and 3′ ends covalently joined [1]. For many years they were thought to be transcriptional background but with the advances of high-throughput sequencing they were identified as stable transcripts in the cell. They are often expressed in a tissue-specific manner and can be dysregulated in complex diseases, such as breast cancer [1,2,3,4].

Breast cancer is one of the most incident types of cancer in women around the world and one of the leading causes of death in women, representing a major health concern worldwide [5]. It can be subdivided in four molecular subtypes: Luminal A, Luminal B, HER-2 enriched, and triple-negative breast cancers (TNBC), each having its own associated risk factors and clinical outcome [6,7]. TNBC is one of the molecular subtypes that has very aggressive behavior and poor prognosis for the patient. Such aggressiveness is usually associated with a high relapse rate and poor survival rates after the first metastatic event [8]. Since circRNAs have a tissue-specific expression profile that can be altered in cancers, Ju et al. were able to predict disease-free and overall survival in colon cancer patients using a classifier based on the expression of four circRNAs [9], indicating that they can be good prognostic biomarkers for other types of cancer, such as TNBC.

CircRNA’s dysregulated expression profile has been observed in several types of cancer, including breast cancer, and correlated with altered physiological roles such as cell proliferation, differentiation, invasion, and apoptosis [10,11,12]. In breast cancer, studies using either microarray [13] or RNA-Seq [4] revealed the general circRNAs dysregulated expression profile, regardless of the molecular subtypes of the disease. It was possible to observe that (i) upregulated circRNAs enriched to different molecular functions when compared to downregulated ones [13] and that (ii) knockdown of important circRNAs (such as *circCNOT2*) significantly reduced the viability of MCF-7 and BT-474 breast cancer cell lines [4].

One of many described functions of circRNAs is the ability to act as microRNAs (miRNAs) sponges, consequently affecting their target gene expression [14,15]. In TNBC, circGFRA1 acts as miR-34a sponge and the overexpression of this circRNA leads to in-creased proliferation rates and was correlated with poor survival of patients [16]. Another study showed that circANKS1B was upregulated in TNBC and sponged miR-148a-3p and miR152-3p, which lead to an increased USF1 expression and ultimately activation of the TGF-ß1/Smad pathway to promote epithelial-to-mesenchymal transition (EMT) [17].

Despite acting as an miRNA sponge is the most studied function of circRNAs, it may not be their main function since circRNAs do not contain more miRNA binding sites as it would be expected by chance [18]. For a single competitive endogenous RNA (ceRNA) acting as an miRNA sponge and having a consequential effect on target genes, a high density of miRNA binding sites is necessary (in hepatocytes, it was an order of 1.5 × 10^5^) [19]. Thus, it is unlikely that circRNAs’ main function is to sponge and regulate miRNA expression.

Another circRNA function, although less studied, is the ability to interact with RNA binding proteins (RBPs), acting either as sponges or scaffolds for the proteins [20,21]. Okholm et al. [22] showed that circRNAs interact with RBPs in a cell-type specific manner and that circularizing exons are enriched with RBP binding sites, suggesting the regulatory activity of the circRNA–RBP interaction.

The global profiling of circRNAs has been studied in breast cancer and was able to distinguish estrogen receptor status between patients [4]; however, there is no global profiling specifically focusing a specific molecular subtype. Here, we aimed to characterize the global expression profile of circRNAs in TNBC, in order to identify potential biomarkers characteristic to this aggressive subtype. Additionally, we investigated in silico their biological function through the interaction with RBPs.

## 2. Materials and Methods

### 2.1. Ethics Approval and Consent to Participate

The study including all experimental protocols was approved by the Ethics Committee of the Center of Tropical Medicine from the Federal University of Pará (No. 043/2008-CEP/NMT) and AC Camargo Cancer Center (No. 1746/13C). All study participants or their legal guardian provided informed written consent in accordance with the Helsinki Declaration. The methods were performed in accordance with the approved guidelines.

### 2.2. TNBC Sample Series, RNA-Seq Data, circRNA and mRNA Mapping, and Data Analysis

In total, 37 triple-negative breast cancer and 25 matched adjacent frozen tissues were obtained from biobank at A.C. Camargo Cancer Center. All cases were previously screened for *BRCA1* and *BRCA2* germline pathogenic variants and the *BRCA1* epigenetic silencing was assessed in tumor DNA by promoter methylation analysis [23]. RNAseq data was generated using TruSeq Stranded Total RNA kit (Illumina, catalog #20020598) and sequenced on Illumina NextSeq500 and RNAseq data was deposited in NCBI SRA (Access number: PRJNA808398). The resulting reads were trimmed using fastp [24] and aligned to the hg19 reference human genome using STAR [25]. Mapped reads were then counted using three different circRNA identification tools: CircExplorer2 [26], CIRI [27], and DCC [28]. CircRNAs that were identified by at least two software were considered for further analysis. In order to evaluate gene expression in the studied samples, mapped reads were also quantified using Salmon [29].

Differential expression analyses were performed using DESeq2 [30] and circRNAs or genes that had an absolute log_2_ Fold Change ≥ 1.5 and an adjusted *p*-value < 0.05 were considered to be dysregulated in TNBC.

### 2.3. CircRNA-RBP Prediction

RNA binding proteins binding sites in the identified differentially expressed circRNAs were predicted and mapped using Circular RNA Interactome online tool (https://circinteractome.nia.nih.gov, accessed on 1 February 2022) [31]. Such database considered over 90 independent CLIP-Seq datasets from various RBPs in order to create a comprehensive catalog of circRNA–RBP interactions.

### 2.4. Functional Enrichment Analysis

Enrichment analysis of the RBPs that interacted with the differentially expressed circRNAs were conducted in KEGG and Reactome pathways using ClusterProfiler [32] and ReactomePA [33] packages in R (ver 4.1.0). All graphs were made using R (v.4.1.0) and interaction networks and enriched pathways were constructed using cnetplot() function in R or Cytoscape (v.3.7.1). Enriched terms with an FDR adjusted *p*-value < 0.05 were considered to be statistically significant.

### 2.5. Biological Samples

We investigated two different sets of TNBC patient samples to validate our results: FFPE and frozen tissues. Samples were obtained from patients undergoing breast biopsy by mammoplasty or surgical resection in Ophir Loyola Hospital (Belém, Brazil). Patients with TNBC diagnosis had their samples collected before undergoing chemotherapy or radiotherapy. All samples were analyzed by a pathologist that confirmed the positive or negative diagnosis of cancer.

FFPE samples were obtained from University Hospital João de Barros Barreto (HUJBB) from the Federal University of Pará (UPFA) and consisted of a total of 23 patients, being 6 without diagnosis of cancer and 17 with TNBC. Frozen tissue samples consisted of 17 samples, 10 being from patients without cancer and 7 from TNBC patients.

### 2.6. Total RNA Isolation and RT-qPCR

Total RNA was isolated from frozen tissues using TRIzol reagent (ThermoFisher, Waltham, MA, USA, catalog #15596018) and from FFPE tissues using High Pure miRNA Isolation Kit (Roche Applied Science, Penzberg, Germany, catalog #5080576001), all according to manufacturer’s instructions. CDNA synthesis was performed with at least 200 ng of RNA input and random hexamers using GoTaq^®^ 2 step RT-qPCR Systems (Promega, Madison, WI, USA, catalog #A6010).

Quantitative real-time PCR was conducted in an ABI Prism 7500 system (ThermoFisher, Waltham, MA, USA) using GoTaq^®^ 2 step RT-qPCR Systems (Promega, Madison, WI, USA, catalog #A6010). Reactions consisted of 10 ng of cDNA, 250 nM of each forward and reverse primers, and 5 µL of qPCR master mix in thermal cycling conditions provided by the manufacturer. Primers utilized are listed in Supplementary Table S1. Expression levels were normalized to the most stable and less variable housekeeping gene, *PUM1* being utilized in frozen tissues and *ACTB* in FFPE tissues. All qPCR experiments were conducted in triplicates.

### 2.7. Statistical Analysis

Normalized expression values were calculated using the Comparative Ct method [34]. Shapiro–Wilk test was used to verify if the normalized expression values followed a Gaussian distribution and Student’s *t*-test was used to compare means between each condition. *p*-values < 0.05 were considered to be statistically significant.

In order to verify if circRNAs expression was able to distinguish TNBC patients from control patients without cancer, Receiver Operating Characteristic (ROC) curves and Area Under the Curve (AUC) were calculated using the pROC package and circRNAs that showed an AUC > 0.75 were considered to be good potential biomarkers. All tests and graphs were performed in R statistical software (ver. 4.1.0).

## 3. Results

### 3.1. CircRNAs and Their Host Genes Are Predominantly Downregulated in TNBC

We analyzed a total of 37 triple-negative breast cancer patients and 25 matched adjacent tissues, resulting in a total of 62 samples. Clinical characteristics and *BRCA* mutational status of the studied patients are presented in Table 1.

**Figure 1:**
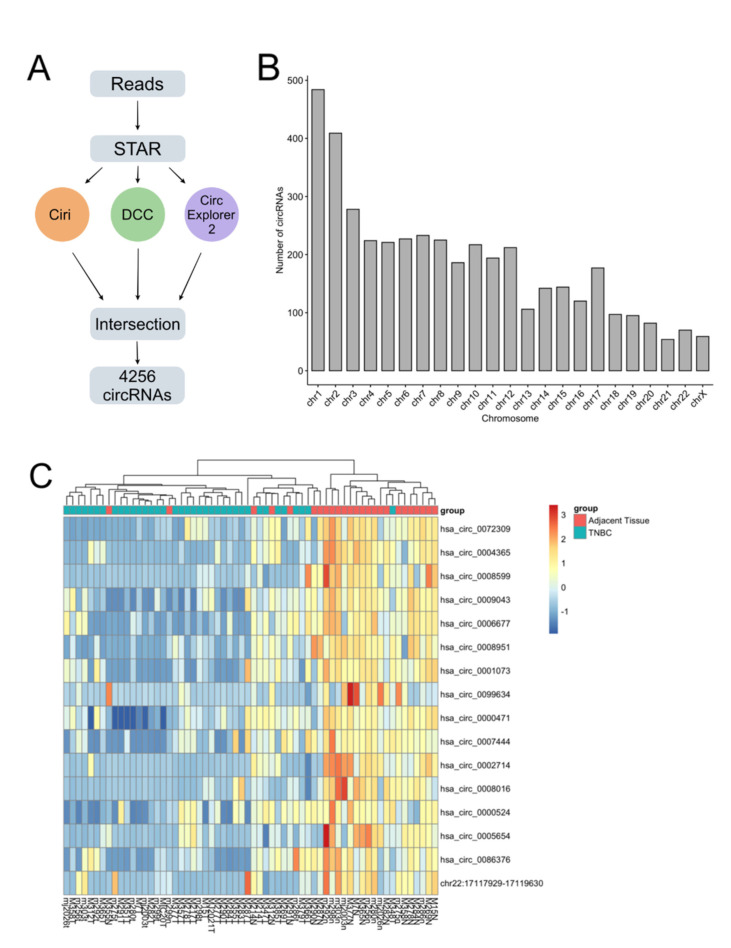
Characterization of circular RNAs (circRNAs) expressed in triple-negative breast cancer (TNBC). (**A**) Schematic overview of circRNA identification protocol, when we only considered circRNAs identified by all three software; (**B**) genomic origin of all identified circRNAs; (**C**) heatmap showing all 16 downregulated circRNAs in TNBC.

In order to confidently identify the circRNAs present in TNBC, we quantified RNA-Seq reads using three different algorithms: CIRI, DCC, and CircExplorer2. Only circRNAs quantified by at least two software were considered and a total of 4256 circRNAs were identified in TNBC (Figure 1A). These circRNAs were derived from genes distributed across the genome, chromosomes one and two displaying the ones with the highest number of circRNAs (Figure 1B).

Next, we compared the expression between TNBC and their matched normal adjacent to identify the circRNAs related to the carcinogenesis of this molecular subtype and found 16 differentially expressed circRNAs, all downregulated in cancer (Figure 1C). Of these, “chr22:17117929-17119630” has not been previously annotated in CircBase and is a novel circRNA involved in TNBC carcinogenesis.

When we compared the circRNAs predicted by both DCC and CircExplorer2, in addition to the very same 16 circRNAs, we found an upregulated one—hsa_circ_0000479, which was included in our analyses. The list of all differentially expressed circRNAs is presented in Table 2.

After identifying the differentially expressed circRNAs in TNBC, we investigated the expression of its respective host gene. It was possible to observe that host genes are differentially expressed and follow the same dysregulated pattern as their coded circRNAs (Supplementary Figure S1).

### 3.2. CircRNAs Interact with RNA Binding Proteins and Enrich to Cancer-Related Pathways

Since interacting with RBPs is one of the less studied functions of circRNAs and is possible to assess it using CLIP-Seq data, we mapped the RBPs that interact with the top five downregulated circRNAs (hsa_circ_0072309, circ_0004365, circ_0006677, circ_0008599, and circ_0009043) and hsa_circ_0000479 using the tool available in CircInteractome.

We observed that the studied circRNAs mapped with a total of 14 RBPs, being hsa_circ_0072309 the one that interacted with the most, mapping to 13 RBPs. The complete list of circRNA–RBP interaction is presented in Table 3. Most of the identified RBPs are proteins that act either in gene regulation (AGO2, FMR1, and LIN28A/B) or gene transcription (ESWR1, FUS, PTBP1, and U2AF), indicating that circRNAs have an important role in mediating such processes.

**Figure 2:**
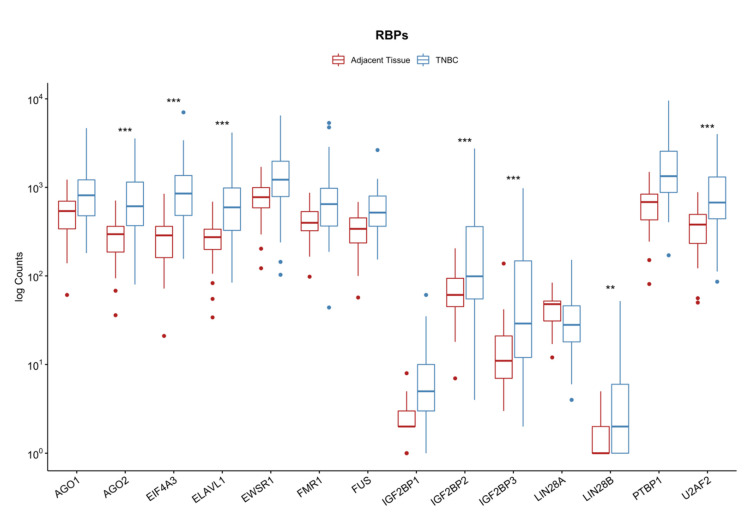
Gene expression of the RNA binding proteins (RBPs) predicted to interact with hsa_circ_0072309, circ_0004365, circ_0006677, circ_0008599, circ_0009043, and circ_0000479. ** *p* < 0.01, *** *p* < 0.001.

Since the dataset used to map RBPs to circRNAs is composed of a large collection of different tissue samples, we evaluated whether the identified RBPs were expressed in our samples and found that all 14 proteins are present in TNBC (Figure 2). From all the RBPs investigated, seven were differentially expressed and upregulated (AGO2, EIF4A3, ELAVL1, IGF2BP2/3, LIN28B, and U2AF2), showing that these genes have an important role in triple-negative breast cancer development.

**Figure 3:**
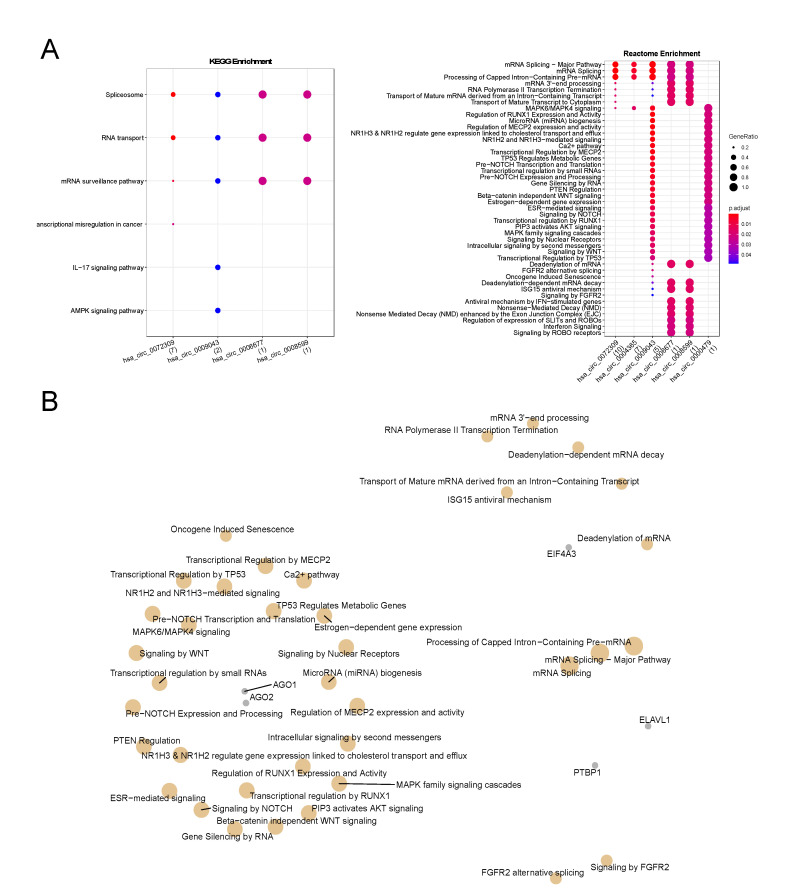
Functional enrichment analysis of the RNA binding proteins (RBPs) bound to hsa_circ_0072309, circ_0004365, circ_0006677, circ_0008599, circ_0009043, and circ_0000479. (**A**) Kyoto Encyclopedia of Genes and Genomes (KEGG) and Reactome enrichments (left and right panels, respectively) for each circRNA that had statistically significant results; (**B**) Gene-Concept network of the Reactome enrichment result showing that AGO1/2, EIF4A3, ELAVL1, and PTBP1 were the main RBPs that enriched in cancer-related pathways.

Next, we performed an enrichment analysis of the mapped RBPs in each circRNA considering both KEGG and Reactome pathways (Figure 3A). We observed that the RBPs that interact with hsa_circ_0004365 and circ_0000479 did not enrich to any KEGG pathway but the others bound to the remaining circRNAs enriched to pathways related to splicing events, RNA transport, and mRNA surveillance. This result indicates that these circRNAs act in important pathways related to gene regulation at the transcriptional level. Additionally, we observed that the RBPs bound to hsa_circ_0009043 also enriched to IL-17 and AMPK signaling pathways, which are important regulators of cell growth, autophagy, cell polarity, and chronic inflammation. Hsa_circ_0072309 was the only one that had its mapped RBPs enriched to pathways related to transcriptional misregulation in cancer, suggesting that this circRNA may be a key element in breast carcinogenesis.

In Reactome enrichment analysis, all circRNAs and their associated RBPs enriched for several pathways related to cancer and gene regulation, such as: microRNA biogenesis, gene silencing by RNA, MAPK family signaling cascades, oncogene induced senescence, PTEN regulation, among others (Figure 3A, right). In order to identify which RBPs were acting in each enriched pathway, we constructed a network to visualize these relations (Figure 3B). We observed that AGO1/2, EIF4A3, ELAVL1, and PTBP1 were the main RBPs acting in most of the cancer-related and gene regulation enriched pathways.

### 3.3. TNBC Patients with BRCA1 Mutations Have a Distinct circRNA Profile When Compared to Wildtype Ones

**Figure 4:**
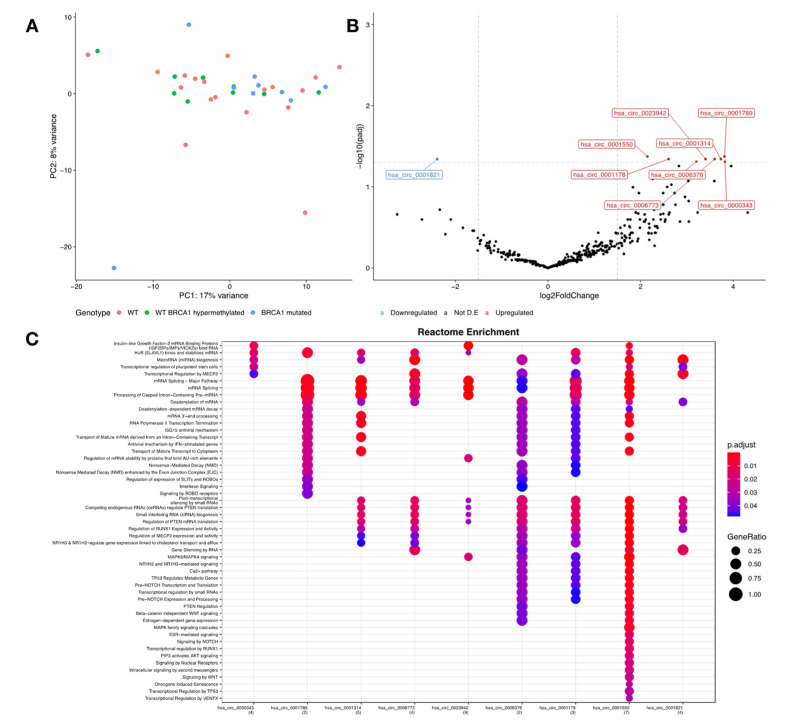
CircRNA transcriptomic profile according to mutational data of TNBC patients. (**A**) Principal component analysis showing overall similarity in expression data between Wildtype, Wildtype with BRCA1 hypermethylated, and BRCA1 mutated patients; (**B**) differential expression analysis showed significant results when comparing Wildtype and BRCA1 mutated patients, in which it was possible to observe nine differentially expressed circRNAs, being one downregulated (hsa_circ_0001821) and eight upregulated (hsa_circ_0001550, circ_0001178, circ_0006376, circ_0023942, circ_0001314, circ_0001789, and circ_0000343); (**C**) Reactome enrichment of the RBPs predicted to interact with these circRNAs revealed similar pathways to the ones enriched in the TNBC vs. adjacent tissue comparison.

As we had the status of *BRCA1* germline pathogenic variant and *BRCA1* somatic epigenetic silencing of the 37 cases, which was previously assessed [23] (Brianese et al., 2018), we were able to evaluate whether *BRCA1* germline or somatic deficiency had an influence on circRNA expression profile. For that, we used the three groups of TNBC samples—WT (sporadic TNBC: negative for *BRCA1/2* germline pathogenic variants (GPV) and non-somatic silencing), WT *BRCA1* hypermethylated (sporadic TNBC: negative for BRCA1/2 GPV and positive for somatic silencing), and *BRCA1* mutated (hereditary or TNBC diagnosed in BRCA1 GPV carriers) and analyzed their transcriptomic data. We observed that there were no major significant differences in circRNA expression among these three groups (Figure 4A), except for WT (sporadic irrespective of somatic *BRCA1* status) vs. *BRCA1* germline mutated, which presented differentially expressed circRNAs (Figure 4B). In this comparison, we observed nine differentially expressed circRNAs, one downregulated (hsa_circ_0001821) and eight upregulated (hsa_circ_0001550, circ_0001178, circ_0006376, circ_0023942, circ_0001314, circ_0001789, and circ_0000343) (Figure 4B). Fold change information and adjusted *p*-values regarding these circRNAs are presented in Supplementary Table S2.

Since there were nine differentially expressed circRNAs in hereditary TNBC, diagnosed in *BRCA1* GPV carriers when compared with sporadic TNBC, we sought to investigate if they interacted with RBPs and, if so, which biological pathways they are involved in. Functional enrichment analysis revealed similar pathways to the ones enriched in the *TNBC* vs. adjacent tissue comparison (Figure 3A), but also enriched in pathways specific to *BRCA1* mutation, such as “Regulation of mRNA stability by proteins that bind AU−rich elements” and “HuR (ELAVL1) binds and stabilizes mRNA” (Figure 4C). The circRNAs hsa_circ_0001550, circ_0001178, and circ_0006376 were the ones that interacted with RBPs that enriched in most of the cancer-related pathways, indicating that these circRNAs and RBPs are key elements in these regulatory networks. In our data, we observed that all predicted RBPs in our analysis were expressed but not differentially expressed when comparing TN tumors of *BRCA1/2* WT patients with TN tumors of *BRCA1* mutated carriers (Supplementary Figure S2), indicating that RBPs have the same expression profile in TNBC regardless of germline *BRCA1* mutational status.

### 3.4. Hsa_circ_0072309 Is a Potential Biomarker of TNBC and May Be Involved in Cancer-Related Gene Regulation Signaling

After observing that hsa_circ_0072309, circ_0004365, circ_0006677, circ_0008599, circ_0009043, and circ_0000479 showed important roles in TNBC carcinogenesis, we sought to validate the potential of these circRNAs as biomarkers in two different sets of independent samples: FFPE and frozen tissues of TNBC and breast without cancer.

**Figure 5:**
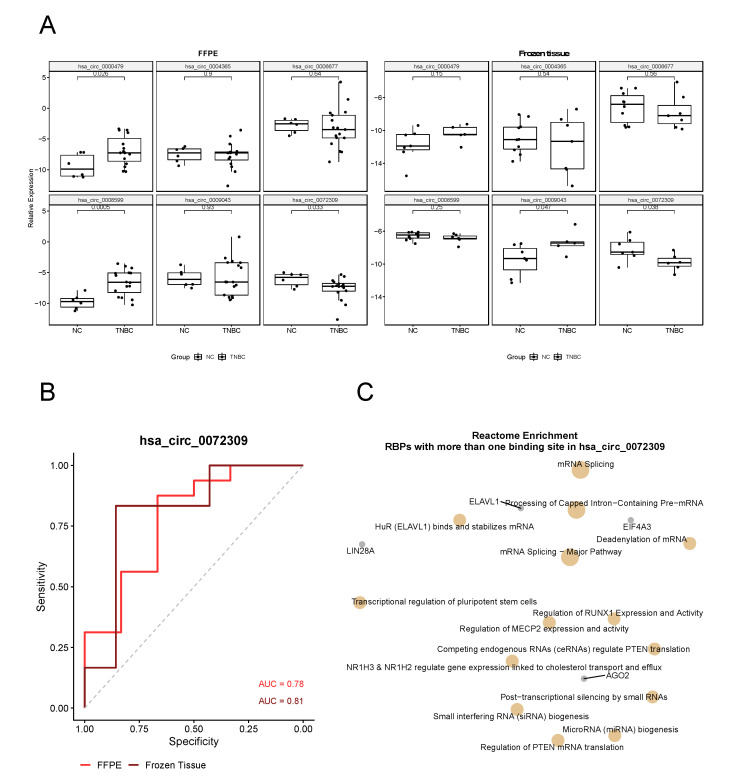
Hsa_circ_0072309 shows a promising potential as a biomarker of TNBC. (**A**) Validation of of hsa_circ_0072309, circ_0004365, circ_0006677, circ_0008599, circ_0009043, and circ_0000479 in two new sample sets: FFPE and frozen tissue (left and right panels, respectively) of TNBC patients (*n* = 6 and *n* = 10, respectively) and patients without cancer (*n* = 17 and *n* = 7, respectively. NC = non-cancer). *p*-values shown are from a Student’s *t*-test. Expression levels were calculated using the Comparative Ct method and ACTB and PUM1 were used as reference genes for frozen and ffpe tissues, respectively; (**B**) ROC curves of hsa_circ_0072309 in FFPE (bright red) and frozen tissue (dark red) samples showing an Area Under the Curve (AUC) with at least 0.78, indicating that this circRNA has good ability in discriminating TNBC from non-cancer patients; (**C**) Reactome enrichment of the RBPs with more than one binding site in hsa_circ_0072309 showing that this circRNA is acting in many cancer-related pathways, such as “Regulation of PTEN translation” and “Regulation of RUNX1 Expression and activity”.

In FFPE samples, we observed that hsa_circ_0000479 and circ_0072309 were differentially expressed correlated to the expression profile observed in our RNA-Seq experiment. However, hsa_circ_0008599 expression profile was different and upregulated in our validation set (Figure 5A). In frozen tissue samples, hsa_circ_0009043 and circ_0072309 were differentially expressed, circ_0072309 being consistent with our RNA-Seq results (Figure 5A).

Since hsa_circ_0072309 was consistently downregulated in all our analyses, we investigated its potential as a biomarker of TNBC using Receiver Operating Characteristic (ROC) curves and Area Under the Curve (AUC) analysis (Figure 5B). It was possible to observe that in both FFPE and frozen tissues, hsa_circ_0072309 expression was able to distinguish TNBC patients from patients without cancer with an AUC of 0.78 and 0.81, respectively.

hsa_circ_0072309 being a potential biomarker of TNBC, we decided to perform a functional enrichment with only the RBPs containing more than one binding site in this circRNA to understand its biological functions better. Only Reactome pathways enriched for those genes, and it was possible to observe that they are involved in important cancer-related pathways such as regulation of *PTEN* translation and regulation of MECP2/RUNX1 expression and activity (Figure 5C).

## 4. Discussion

Circular RNAs have been in the spotlight in the past in biomedical research, especially because they are differentially expressed in many complex diseases including cancer and do not have a clearly established biological function [52,53,54,55]. More than 90,000 circRNAs have been identified in humans but only 1% of them have a biological function described [56].

The global circRNA expression profiling has been realized in primary breast cancer before [4,57], but no study focused on analyzing a molecular subtype in particular has been performed. We evaluated the circRNAome of triple-negative breast cancer, a very aggressive subtype of breast cancer, with high tendency of metastasizing and poorer rates of survival [8].

In our analysis, we observed a total of 4256 distinct circRNAs in TNBC and in paired adjacent tissues, that originated from all chromosomes (Figure 1B). Of all circRNAs, 17 were differentially expressed, 16 being downregulated and 1 being upregulated (Table 2).

From the six most differentially expressed circRNAs (hsa_circ_0072309, circ_0004365, circ_0006677, circ_0008599, circ_0009043, and circ_0000479), only circ_0072309, circ_0009043, and circ_0000479 have been identified in complex diseases [58,59,60,61,62].

Hsa_circ_0072309 was found to be downregulated in renal and breast carcinomas [61,62], and circ_0009043 was described as downregulated in endometrial carcinoma [59]. Circ_0072309 is supposed to act as an miR-492 sponge in breast cancer cells and its downregulation leads to increased proliferation, migration, and invasion rates [62]. In renal carcinoma, circ_0072309 sponged miR-100 and a downregulation of this circRNA caused miR-100 increased activity which leads to activation of PI3K/AKT and mTOR pathways, important cancer-related pathways [61].

Even though the most the described circRNA function is being microRNA sponges, the interaction of circRNA–RBPs has been described to have potential regulatory roles. A recent study by Okholm et al. [22] demonstrated that some RBPs preferentially bind to circRNAs rather than their linear counterpart and that such interaction occurs in a cell-type specific manner. They also observed that circCDYL interacts with IGFBP1 and IGFBP2 in bladder cancer cell lines and that depletion of either circCDYL or those RBPs was a hallmark of cancer gene sets and knockdown of this circRNA affected the expression of *TP53* and *MYC*, two important genes associated with tumor progression [22].

Since interacting with RBPs is a relevant function of circRNAs in cancer, we decided to investigate if hsa_circ_0072309, circ_0004365, circ_0006677, circ_0008599, circ_0009043, and circ_0000479 were interacting with such proteins. We observed that these six circRNAs interact with 14 RBPs that have major functions within the cell, such as gene regulation, splicing events, mRNA stability, RNA transport, and translation (Table 3). Of all circRNAs, hsa_circ_0072309 interacted with 13 RBPs and most of them either act in gene regulation in both post-transcriptional and translational levels.

Overall, functional enrichment of these 14 RBPs revealed their association to several mRNA-related processes, including splicing and transport of mature mRNAs, but also to well-known cancer signaling pathways such as transcriptional regulation by TP53 (Figure 3). Interestingly, we also found an enriched estrogen-dependent gene expression pathway, which is a breast-cancer-related process. The regulatory network showed AGO1/2, EIF4A3, ELAVL1, and PTBP1 as hub elements, suggesting that these RBPs may be pivotal in breast carcinogenesis by participating in the control of gene expression (Figure 3 and Table 3).

Noticing that these six circRNAs are important elements in the network of gene regulation in TNBC, we decided to evaluate their expression profile in another two different sets of samples (FFPE and frozen tissues) in order to evaluate if they can be revealed as novel potential biomarkers of TNBC. When performing qRT-PCR of the six studied circRNAs, we observed that in FFPE samples hsa_circ_0072309, circ_0008599, and circ_0000479 were differentially expressed and in frozen tissue samples hsa_circ_0072309 and circ_0006677 had different expression profiles between TNBC and normal breast samples (Figure 5A).

Only hsa_circ_0072309 had a consistent expression profile in these two sample sets and was concordant with our RNA-Seq analysis. This may have occurred because we used normal breast samples, obtained from patients without cancer, in our validation set instead of adjacent-to-tumor as it was in the RNA-Seq. It has been shown that the adjacent-to-tumor tissue is not completely normal tissue and shares some of the molecular alterations already present in the tumor despite being histologically normal [63]; such fact is known as field cancerization. Field cancerization has been shown to influence circRNA expression profiles in gastric cancer [55] and such molecular mechanism may be underway in TNBC, but more studies are necessary to fully understand field cancerization and circRNA expression in breast cancer.

Since hsa_circ_0072309 was the circRNA that was differentially expressed in all our analyses, we decided to evaluate its potential as a risk biomarker of TNBC. When performing ROC curves and AUC analysis, we observed that its expression was able to properly distinguish TNBC from control samples with an AUC of 0.78 and 0.81 for FFPE and frozen tissues, respectively (Figure 5B), indicating that it has good potential of being a risk factor for TNBC.

To further investigate the role of hsa_circ_0072309 in TNBC, we realized a functional enrichment in its predicted RBPs. Functional enrichment analysis of its RBPs target showed some interesting pathways, mostly related to transcriptional and post-transcriptional control of gene expression (Figure 5C). Among them, LIN28A is involved in the pathway of transcriptional regulation of pluripotent stem cells and was previously described in breast cancer as a regulator of multiple tumor-associated progressions, such as proliferation, chemo-resistance, metabolism, inflammation, stemness, and cell development [64].

Curiously, we found nine differentially expressed circRNAs between WT and *BRCA1* mutated patients (Figure 4B). It suggests that mutations in *BRCA1* not only have consequences related to the loss-of-function of itself, but also epigenetic effects by affecting circRNAs’ expression somehow. In this case, functional enrichment of the RBPs by these nine circRNAs showed that *BRCA1* mutations may interfere in major pathways related to carcinogenesis (Figure 4C).

Our study highlighted the importance that circRNAs have in TNBC, identified hsa_circ_0072309 as a novel potential risk biomarker, and identified possible pathways in which these circRNAs can be acting and are associated with TNBC carcinogenesis. Recognizing these differentially expressed circRNAs and identifying their role can lead to a better understanding of deregulated pathways in TNBC and ultimately develop personalized therapies in this molecular subtype of breast cancer.

## 5. Conclusions

Our study highlighted the importance circRNAs have in TNBC and showed potential pathways in which these circRNAs can be acting and are associated with TNBC carcinogenesis. We also identified hsa_circ_0072309 as a novel potential risk biomarker of TNBC. Recognizing these differentially expressed circRNAs and identifying their role can lead to a better understanding of dysregulated pathways in TNBC and ultimately allow the development of personalized therapies in this molecular subtype of breast cancer.

## Figures and Tables

**Table 1:** Clinical characteristics and outcome of TNBC patients.

Clinical Characteristics		Wildtype	Wildtype, *BRCA* Hypermethylated		*BRCA1* Mutation	
		*n* = 18	*n* = 10	*p*-Value *	*n* = 9	*p*-Value *
Age of onset, mean (SD)		41.8 (6.1)	38.5 (7.6)	0.3	36.5 (5.6)	0.16
		nº (%)	nº (%)		nº (%)	
TNM classification, T	T1	3 (16.7)	-	0.11	1 (11.1)	0.94
	T2	8 (44.4)	9 (90)		5 (55.6)	
	T3	3 (16.7)	1 (10)		2 (22.2)	
	T4	4 (22.2)	-		1 (11.1)	
TNM classification, N	N0	5 (27.8)	8 (80)	0.054	5 (55.6)	0.59
	N1	7 (38.9)	2 (20)		3 (33.3)	
	N2	4 (22.2)			1 (11.1)	
	N3	2 (11.1)			-	
TNM classification, M	M0	11 (61.1)	8 (80)	0.41	4 (44.4)	0.06
	M1	7 (38.9)	2 (20)		2 (22.2)	
	Mx	-	-		3 (33.3)	
Tumor stage	I	2 (11.1)	-	0.07	1 (11.1)	0.45
	II	7 (38.9)	9 (90)		5 (55.6)	
	III	7 (38.9)	1 (10)		1 (11.1)	
	IV	2 (11.1)	-		-	
Tumor grade	2	4 (22.2)	-	0.26	-	0.056
	3	14 (77.8)	10 (100)		7 (77.8)	
Chemotherapy	No	11 (61.1)	8 (80)	0.5	6 (66.7)	0.65
	Yes	4 (22.2)	2 (20)		3 (33.3)	
TP53 mutation	No	4 (22.2)	-	0.13	4 (44.4)	1
	Yes	12 (66.7)	6 (60)		5 (55.6)	
Family history	No	10 (55.6)	5 (50)	0.87	2 (22.2)	0.001
	Yes	2 (11.1)	2 (20)		7 (77.8)	
Outcome						
Relapse or metastasis	No	11 (61.1)	5 (50)	0.69	7 (77.8)	1
	Yes	7 (38.9)	5 (50)		2 (22.2)	
Death	No	13 (72.2)	7 (70)	1	7 (77.8)	1
	Yes	5 (27.8)	3 (30)		2 (22.2)	

SD: standard deviation; TP53: tumor protein p53. * *p*-values were calculated using Fisher’s Exact test for categorical variables and a pairwise *t*-test (with FDR correction after multiple comparisons) for continuous variables (age). Statistically significant values (*p* < 0.05) are highlighted in bold.

**Table 2:** Differentially expressed circRNAs in TNBC.

Host Gene	Strand	circRNA	BaseMean	Log2FC	Padj
*LIFR*	−	hsa_circ_0072309	7.478	−2.392	4.68 × 10^−11^
*SEMA3C*	−	hsa_circ_0004365	5.284	−2.347	6.61 × 10^−10^
*MIR31HG*	−	hsa_circ_0008599	3.758	−1.981	3.61 × 10^−06^
*EXOC6B*	−	hsa_circ_0009043	10.768	−1.955	2.74 × 10^−07^
*WDR78*	−	hsa_circ_0006677	8.343	−1.931	1.29 × 10^−07^
*FAM126A*	−	hsa_circ_0008951	4.761	−1.925	2.74 × 10^−07^
*ACVR2A*	+	hsa_circ_0001073	11.392	−1.895	3.01 × 10^−07^
*RMST*	+	hsa_circ_0099634	3.223	−1.846	8.57 × 10^−05^
*N4BP2L2*	−	hsa_circ_0000471	24.928	−1.825	3.61 × 10^−06^
*RHOBTB3*	+	hsa_circ_0007444	8.915	−1.700	1.66 × 10^−04^
*SEMA3C*	−	hsa_circ_0002714	2.767	−1.693	2.00 × 10^−04^
*FGFR1*	−	hsa_circ_0008016	2.983	−1.641	3.11 × 10^−04^
*RBM23*	−	hsa_circ_0000524	7.974	−1.626	8.57 × 10^−05^
*PRDM5*	−	hsa_circ_0005654	2.864	−1.584	5.28 × 10^−04^
*NFIB*	−	hsa_circ_0086376	7.423	−1.540	2.00 × 10^−04^
*TPTEP1*	+	chr22:17117929-17119630	5.560	−1.522	2.76 × 10^−03^
*EPSTI1*	−	hsa_circ_0000479 *	4.422	1.560	2.55 × 10^−04^

* Predicted by DCC and CircExplorer2. Strand = − (antisense) and + (sense); CircRNA = circular RNA; Log2FC = Log2 Fold Change; padj = adjusted *p*-value.

**Table 3:** RBPs predicted to bind in the studied circRNAs and their described functions.

circRNAs	RBP	Number of circRNAs Binding Sites *	RBP Function	Reference
hsa_circ_0009043	AGO1	1	miRNA-mediated gene regulation	[35]
hsa_circ_0072309, hsa_circ_0009043, hsa_circ_0004365, hsa_circ_0000479	AGO2	3/1/6/1	miRNA-mediated gene regulation	[35]
hsa_circ_0072309, hsa_circ_0009043, hsa_circ_0004365, hsa_circ_0008599, hsa_circ_0006677	EIF4A3	6/4/6/1/2	RNA splicing by acting as a core component of the spliceosome and splicing-dependent exon junction complex	[36]
hsa_circ_0072309, hsa_circ_0009043, hsa_circ_0004365	ELAVL1 (HuR)	2/2/2	Increase mRNA stability through binding to their 3’-UTR	[37]
hsa_circ_0072309	EWSR1	4	Regulating transcription through interaction with CREB-binding protein; RNA splicing by cooperating with multiple splicing factors	[38,39]
hsa_circ_0072309	FMR1 (FMRP)	3	Associates in an RNA-dependent manner with MOV10 and facilitates miRNA-mediated gene silencing; binds to mRNAs and mediates RNA transport from nucleus to cytoplasm	[40,41]
hsa_circ_0072309	FUS	1	Mediates the binding of U1 snRNP and RNAPII, being required for splicing to occur during transcription	[42]
hsa_circ_0072309, hsa_circ_0004365	IGF2BP1	1/1	Binding to cytoplasmic mRNAs in order to prevent premature RNA decay; transport RNA in the cytoplasm and provide stability to bound mRNAs	[43,44,45]
hsa_circ_0072309, hsa_circ_0004365	IGF2BP2	1/1	Binding to cytoplasmic mRNAs in order to prevent premature RNA decay; transport RNA in the cytoplasm and provide stability to bound mRNAs	[43,44,45]
hsa_circ_0072309, hsa_circ_0004365	IGF2BP3	1/2	Binding to cytoplasmic mRNAs in order to prevent premature RNA decay; transport RNA in the cytoplasm and provide stability to bound mRNAs	[43,44,45]
hsa_circ_0072309	LIN28A	2	Stimulates translation by actively recruiting RNA helicase A to polysomes; inhibiting miRNA biogenesis	[46,47]
hsa_circ_0072309	LIN28B	1	Stimulates translation by actively recruiting RNA helicase A to polysomes; inhibiting miRNA biogenesis	[46,47]
hsa_circ_0072309, hsa_circ_0009043, hsa_circ_0004365	PTBP1	1/1/3	Exon exclusion during alternative splicing events and mRNA stabilization; stimulates translation at picorna virus internal ribosome entry sites (IRES)	[48,49]
hsa_circ_0072309	U2AF2 (U2AF)	1	Activation of splicing and its coupling to transcription; 3’ end processing of vertebrates	[50,51]

* In the order listed, respectively. CircRNA = circular RNA; RBP = RNA binding protein.

## Data Availability

Sequencing data for RNA-Seq are accessible from NCBI’s Sequence Read Archive (SRA) with the accession number PRJNA808398.

## Supplementary Materials

The following are available online at https://www.mdpi.com/article/10.3390/cancers14133280/s1, Figure S1: Differentially expressed circRNAs’ host genes are also dysregulated and follow the same pattern as their coded circRNAs; Figure S2: All RBPs that are predicted to interact with hsa_circ_0001550, circ_0001178, circ_0006376, circ_0023942, circ_0001314, circ_0001789, and circ_0000343 are being expressed in TNBC and when comparing Wildtype with BRCA1 mutated patients, there is no difference in expression regarding mutational status; Table S1: Information and primer sequences utilized in qRT-PCR; Table S2: Differentially expressed circRNAs in TNBC patients with BRCA1 mutations when comparing to Wildtype ones.
