# Supplementary material for: Triple-Negative Breast Cancer circRNAome Reveals Hsa_circ_0072309 as a Potential Risk Biomarker
Source: Cancers (Basel). 2022 Jul 5;14(13):3280. doi: 10.3390/cancers14133280 (PMC9265318; doi:10.3390/cancers14133280)
Supplement: Supplementary file 1 [file cancers-14-03280-s001.zip › cancers-1673528-supplementary.pdf]

cancers

MDPI

Supplementary Materials

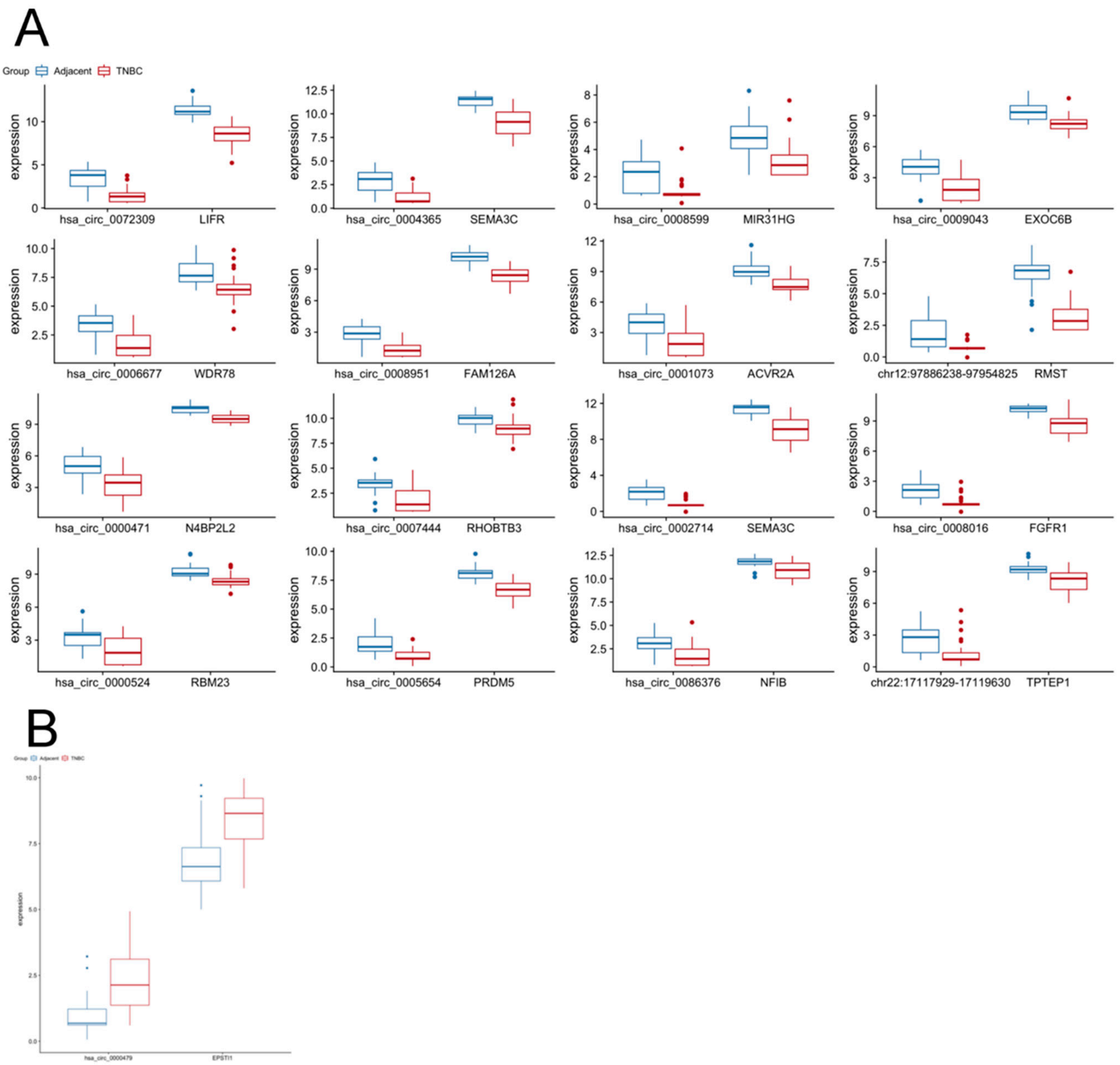

Figure S1. Differentially expressed circRNAs' host genes are also dysregulated and follow the same pattern as their coded circRNAs.

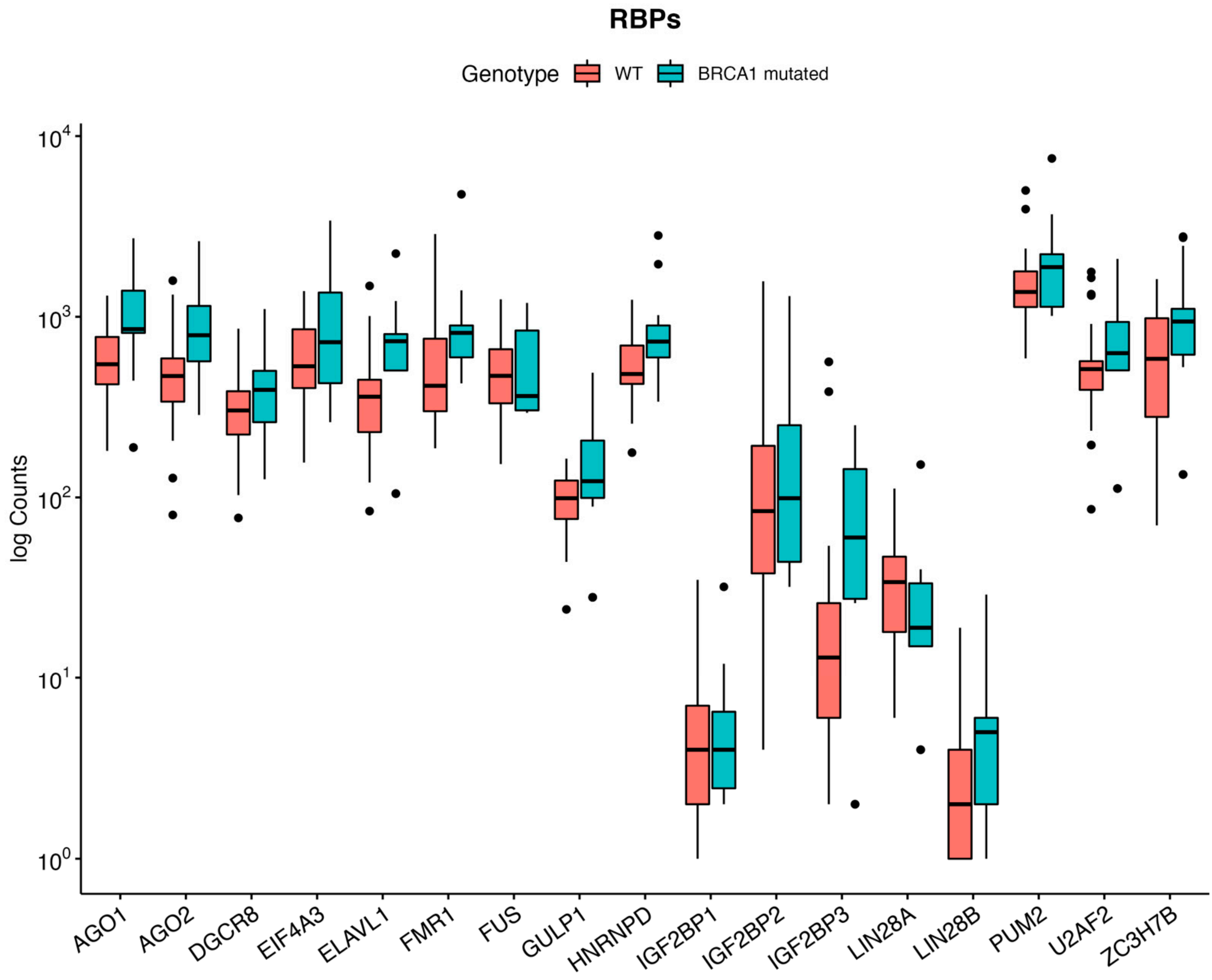

Figure S2. All RBPs that are predicted to interact with hsa_circ_0001550, circ_0001178, circ_0006376, circ_0023942, circ_0001314, circ_0001789 and circ_0000343 are being expressed in TNBC and when comparing Wildtype with BRCA1 mutated patients, there is no difference in expression re-garding mutational status.

Table S1. Information and primer sequences utilized in qRT-PCR.

Primer	Sequence (5'> 3')	Type of primer	Product size
ACTB qPCR F	GCACAGAGCCTCGCCTTT	Converging	70 bp
ACTB qPCR R	TATCATCATCCATGGTGAGCTGG	Converging	
PUM1 qPCR F	ACGTCCTGTCATTGGCACTA	Converging	93 bp
PUM1 qPCR R	TCTCATTAATTACCTGCTGGTCT	Converging	
circ_00072309 qPCR F	GCTCGTAAAATTAGACTGACTGC	Diverging - exon junction	70 bp
circ_00072309 qPCR R	CAGGATGGTCGTTTCAAACATAC	Diverging	
circ_0004365 qPCR F	TCTCGGAACAGGACCAAGTT	Diverging - exon junction	85 bp
circ_0004365 qPCR R	GTGTTCACGTTGGGGTTGAA	Diverging	
circ_0008599 qPCR F	TTCCCAGTTTCAGACCACCC	Diverging	79 bp
circ_0008599 qPCR R	GGACCAGAAACCTGCAGTGT	Diverging - exon junction	
circ_0009043 qPCR F	GCCATGAAGCAAAATCAAGTGA	Diverging - exon junction	72 bp
circ_0009043 qPCR R	TGCTATTACCAGTTCCTTTCCCT	Diverging	
circ_0006677 qPCR F	TAGCAGAAGACCTGGAAGAACCA	Diverging	70 bp
circ_0006677 qPCR R	TGTGGCATTGTTCCTGTGAAACT	Diverging - exon junction	
circ_0000479 qPCR F	AGCATCAGCAATACACAAGTGC	Diverging - exon junction	81 bp
circ_0000479 qPCR R	GCTCCGCAATTCTTTGTATCTC	Diverging	

cancers MDPI

Table S2. Differentially expressed circRNAs in TNBC patients with BRCA1 mutations when comparing to Wildtype ones.

Gene	Strand	CircRNA	Expression Profile	BaseMean	Log2FC	Padj
CLSN1	-	hsa_circ_0000343	Upregulated	2.478855444	3.822544815	0.0489
RAB11FIP1	-	hsa_circ_0001789	Upregulated	3.872956911	3.81113078	0.0422
FAM208A	-	hsa_circ_0001314	Upregulated	1.785044727	3.73400603	0.0454
HIBADH	-	hsa_circ_0006773	Upregulated	1.801634675	3.600076978	0.0454
PICALM	-	hsa_circ_0023942	Upregulated	2.140926545	3.401582269	0.0454
HOOK3	+	hsa_circ_0006376	Upregulated	3.35953751	3.204640961	0.0489
USP25	+	hsa_circ_0001178	Upregulated	4.27055334	2.605783073	0.0454
RARS	+	hsa_circ_0001550	Upregulated	12.40200043	2.146674774	0.0422
TCONS_00015354	+	hsa_circ_0001821	Downregulated	5.494354843	-2.391511722	0.0454
